# Standardization of *Euphorbia tithymaloides* (L.) Poit. (Root) by Conventional and DNA Barcoding Methods

**DOI:** 10.1021/acsomega.3c02543

**Published:** 2023-08-04

**Authors:** Shital Patil, Mohd Imran, R. Sahaya Mercy Jaquline, Vidhu Aeri

**Affiliations:** † Department of Pharmacognosy and Phytochemistry, School of Pharmaceutical Education and Research, Jamia Hamdard, Hamdard Nagar, New Delhi 110062, India; ‡ Department of Pharmacognosy and Phytochemistry, School of Pharmaceutical Education and Research, Jamia Hamdard, Hamdard Nagar, New Delhi 110062, India

## Abstract

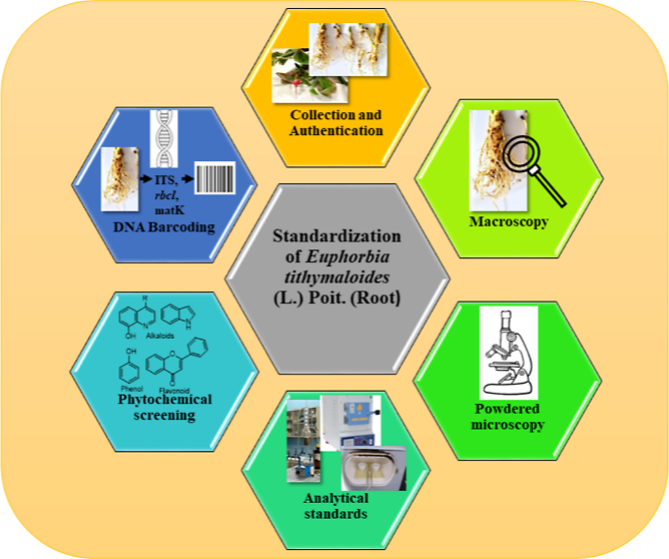

Adulteration and substitution of medicinal plants have become a matter of great concern in recent years. *Euphorbia tithymaloides* is one such medicinal plant that has gained importance but is often confused with other plants of the same species. In order to address this issue, this study aimed to conduct a conventional and molecular pharmacognostic study for the identification of the root of *E. tithymaloides*. The root of the plant was studied for the macroscopic observations, and then, the root was ground into coarse powder for microscopic studies and to determine the physiochemical properties. The powder was subjected to extraction with solvents such as ethanol, ethanol/water (1:1), hexane, and ethyl acetate. The extracts were then used for qualitative and quantitative (phenol, alkaloids, and flavonoids) phytochemical analysis. The molecular study was performed with the DNA barcoding technique. The DNA was extracted from the root of the plant, and its purity was examined by gel electrophoresis (1% w/v). The DNA was then amplified using an Applied Biosystems 2720 thermal cycler for the rbcL, matK, and ITS primers. The amplified primers were sequenced with a 3130 Genetic Analyzer, and the generated sequences were searched for similarity in the GenBank Database using the nucleotide BLAST analysis. The micro- and macroscopic studies revealed the morphological and organoleptic characters as well as the presence of medullary rays, fiber, cork, sclereids, parenchymal cells, and scalariform vessels. The physiochemical properties were found within the limit. The phytochemical analysis revealed the presence of terpenoids, flavonoids, saponins, and alkaloids. In addition, the alkaloidal content was high in the ethanol extract (63.04 ± 3.08 mg At E/g), while the phenol content was high in the hexane extract (10.26667 ± 1.77 mg At E/g), and the flavonoid content was high in the ethyl acetate extract (41.458 ± 1.33 mg At E/g). After the BLAST analysis from the GenBank database, the rbcL, ITS, and matK primers showed a similarity percentage of 99.83, 99.84, and 100. The phylogenetic tree for the species closest to each primer was generated using the MEGA 6 software. The matK loci had the highest percentage similar to the rbcL and ITS loci, indicating that the matK loci can be used to identify the root of *E. tithymaloides* as a standalone. The results from this study can be used to establish a quality standard for *E. tithymaloides* that will ensure its quality and purity.

## 1 Introduction

The use of medicinal plants for treating various illnesses and disorders has a long history, stretching back centuries. In recent years, the use of medicinal plants in modern medicine has seen a rapid increase due to their potential benefits in treating conditions like cancer, chronic pain, and other prevalent health problems.^1^ The efficacy of medicinal plants in therapies warrants further investigation, but the encouraging outcomes of early studies indicate that many of these plants may have a significant impact on healthcare. This might rapidly increase the production of medicinal plant-based products such as teas, tinctures, ointments, and other forms of medicine, which will become more accessible to consumers, leading to improved health outcomes for many individuals. This is important for a variety of reasons, such as being more cost-effective than other medicines, being organic and natural, and being readily available in many parts of the world. In addition, these plants often have fewer side effects than other treatments especially synthetic drugs and heavy metal formulations.^2^

As the medicinal plant industry continues to expand with better profits, substitution and adulteration of herbal ingredients have become more and more of an issue. These practices are documented in ancient medical texts, such as the Ebers Papyrus,^3^ as well as in more recent sources like the British Pharmacopoeia.^4^ Authentication and identification of herbal ingredients are necessary to address the problem of adulteration and substitution. With time, plants have become increasingly important to human medical treatments and advancements. The extinction of certain medicinal plants can have a drastic effect on the continued growth of medical treatments, leading to a greater understanding of the importance of these plants. Without them, it is impossible to make progress in many areas of medical research. The regulation of medicinal plants is a very important and difficult task, which is why it is necessary to have stringent laws to ensure safety and quality and to distinguish between the adulteration and substitution of medicinal plants.^5^ Despite increased regulations, it is hard to guarantee the safety and quality of herbal products. This not only affects the people consuming these plants but also adversely affects the environment as well. The major part of quality control (QC) is to check the product for any adulteration or contamination to determine its safety and efficacy. Generally, QC is done with analytical techniques, which involve the use of electrophoretic,^6^ chromatographic (HPLC, GC, and SCF),^7^ and hyphenated methods (ICPMS, fluorescence),^8^ including specific and non-specific detector systems. Some other aspects that are included in QC are physical examination, microscopical examination, chemical examination, microbiological examination, stability testing, toxicological examination, and a combination of the aforementioned techniques.^9^ Despite these advances, adulteration and substitutions persist in the current period. These conventional pharmacognostic studies and techniques, even the use of modern hyphenated analytical systems, also failed to detect adulterants and substitutions.

On the other hand, molecular biology has had a significant impact on plant science research, allowing us to uncover many previously unknown genetic and behavioral traits in plants. These genetic traits are very helpful in identifying plants of particular species in cases of adulteration and substitution.^10,11^ There are several techniques available, including conventional sequencing, non-Sanger sequencing, random amplifiable polymorphic DNA, DNA barcoding, amplifiable fragment length polymorphism, microsatellites, single nucleotide polymorphism, and others. The limitation with a few of these methods is that no ideal marker exists for all the species, and sometimes, it may mislead in identifying the taxonomy but could be highly beneficial in population genetics.^12^ Because of its accuracy and reliability to identify variations, DNA barcoding has gained the most popular of all methods. Traditional methods of plant identification, such as morphological analysis, can be subjective and prone to error, particularly in cases where species are closely related or have similar physical characteristics.^13,14^ On the other hand, DNA barcoding enables accurate identification of a plant species based on its distinct genetic make-up. Since every plant has a different DNA sequence, it includes using a short, standardized DNA sequence as a “barcode” to identify a certain species of plant. The chloroplast gene rbcL, matK, and trnH-psbA are the most frequently used barcode region, but the ITS (Internal Transcribed Spacer) marker has been also used in recent studies.^15,16^ In a study, the ITS marker was used for DNA barcoding of *Euphorbia*species, and the researchers found that this marker was able to effectively distinguish between *Euphorbia* subgenus and closely related species. It was reported that the ITS marker might be used to identify *Euphorbia* species and to verify the authenticity of the plant material used in conventional medicine and the pharmaceutical industry.^17^ Similarly, Akilabindu in 2019 used chloroplast rbcL and matK gene from *Flacourtia inermis* Roxb for barcoding. It was reported that rbcL gene showed 100% similarity and matK gene showed 99.20% similarity with *Flacourtia jangomas*.^18^ These studies strongly suggest that matK, ITS, and rbcL markers are intensively used in plant DNA barcoding. Despite being a promising technique, employing DNA barcoding for the authentication and identification of medicinal plants comes with a number of limitations such as limited reference libraries, inter- and intra-specific variation, and quality of plant material, cost, and complexity.^19^

*Euphorbia tithymaloides*, also known as “devil’s-backbone” or “coast spurge,” is a medicinal plant that belongs to the family Euphorbiaceae. It is widely distributed in Central and South America, including the African and Caribbean. The plant is found in many other subtropical and tropical areas as an invasive species. The plant is traditionally used in folk medicine to treat various ailments, such as inflammatory conditions, fever, and tumors. Some studies have also shown that extracts of *E. tithymaloides* have pharmacological activities such as anti-inflammatory, anti-tumor, anti-diabetic, anti-cancer, anti-leishmanial, anti-malarial, anti-helminthic, anti-microbial, anti-oxidant, anti-ulcerogenic, and cytotoxicity.^20,21^ Extensive and elaborative research works have been carried out in the aerial parts of the plant, while the roots are yet to be explored. Hence, this study aimed to carry out the pharmacognostical study of *E. tithymaloides* root by both conventional and molecular methods.

## 2 Materials and Methods

### 2.1 Collection and Authentication of Plant

In March 2022, root parts of *E. tithymaloides* were collected from Jamia Hamdard Herbal Garden, New Delhi, India. Department of Botany, Jamia Hamdard, New Delhi, India, helped to identify and authenticate the plant. For future reference, a sample of the plant material was deposited in the herbarium under the voucher specimen number BOT/DAC/2022/01. The roots were cleaned with water to remove mud, broken into small pieces, and completely air-dried. The powder was then pulverized into a coarse powder using an analytical milling machine and stored in an airtight glass jar for use in the current work.

### 2.2 Macroscopic Examination of Root

The fresh roots of *E. tithymaloides* were examined using visual perception. The color, odor, and taste of the root were observed and recorded as organoleptic properties. The macroscopic characteristics of the root, such as shape, size, fracture, and other surface features, were observed using the protocol mentioned in Indian pharmacopeia.^22^

### 2.3 Powdered Microscopy of Root

500 mg of a moderately fine (44/85) grounded root powder was immersed in 10 mL of water (1:20) and left to stand overnight for 24 h. Subsequently, the contents were put into a Petri-plate, and a slide was prepared by placing the contents with a brush on a clean and dry slide. The contents were then examined using a Motic microscope moticam 3.0 MP, AE 2000, and images were taken.

### 2.4 Determination of Analytical Standards

To assess the purity and quality of the crude drug, analytical standards and physicochemical constants of the root were determined. The ash value (water-soluble ash, acid-insoluble ash, and total ash), foaming index, foreign matter, swelling index, moisture content, and extractive value (alcohol-soluble and water-soluble extractives) of root powdered were determined using the standard protocol provided by the Indian Pharmacopoeia^22^ and WHO.^23^

### 2.5 Preparation of Extracts

The extracts were prepared by using the Soxhlet apparatus by increasing the polarity of a solvent such as ethanol/water (1:1), ethyl acetate, hexane, and ethanol. For each solvent, 2 h was given for extraction with optimal temperature.

### 2.6 Phytochemical Analysis

#### 2.6.1 Qualitative Phytochemical Analysis of the Crude Extract

To investigate the existence of different secondary metabolites in the crude extract, qualitative phytochemical assays were carried out using established methods. Saponin test with froth test; terpenoid/steroid test with Liebermann–Burchard reagent; flavonoid test with Shinoda test; Tannin test with ferrous(III) chloride; and alkaloid test with Dragendorff reagents.^24^

#### 2.6.2 Quantitative Phytochemical Analysis of the Crude Extract

The quantitative phytochemical tests were done using standard procedures to detect the number of secondary metabolites such as alkaloid, flavonoid, and phenolic compounds in the crude extract.

##### 2.6.2.1 Determination of Total Flavonoid Content

Total flavonoid content was determined using a slightly modified version of the Sembiring et al. aluminium chloride colorimetric test.^25^ Standard quercetin solutions of 20, 40, 60, 80, and 100 g/mL were produced in 96% ethanol. The standard extract solution (1 mg/mL) was prepared. 12 μL of 10% aluminium chloride solution, 60 μL of extract solution, 180 μL of 96% ethanol, and 12 μL of 1 M sodium acetate were added to the mixture in a 96-well plate. 96 percent ethanol was used as a reagent blank. After being mixed, each substance was incubated for 40 min at room temperature in a dimly lit area. The absorbance at 415 nm was measured with a microplate reader. Total flavonoid content was calculated as quercetin equivalents per gram of plant extract.

##### 2.6.2.2 Determination of Total Phenolic Content

The Folin–Ciocalteu method for a 96-well microplate was optimized based on Sembiring et al.^25^ In a flat-bottom microplate, a standard solution of extracts (1 mg/mL) was mixed with the Folin–Ciocalteu reagent (1:4) in a 40 and 400 μL ratio and shaken for 2 min. After 5 min, sodium carbonate solution (100 g/L) was added (125 μL) and shaken for 1 min at a medium speed. Absorbance was measured at 765 nm using a VersaMax Absorbance Microplate Reader after 3 h at room temperature. The absorbance was corrected by subtracting the ethanol control reaction from the sample reaction. Gallic acid in dilutions of 10, 50, 100, 150, and 200 μg/mL was used as calibration standards. The total phenolic content was expressed in milligrams of gallic acid equivalents (GAE) per gram of plant extract.

##### 2.6.2.3 Determination of Total Alkaloidal Content

The total alkaloid content was determined using the method by Ajanal et al.^26^ Bromocresol green solution was made by dissolving 69.8 mg of BCG in 3 mL of 2 N NaOH and 5 mL of distilled water and then diluted to 1000 mL with distilled water. Phosphate buffer (pH 4.7) was prepared by adjusting the pH of 2 M sodium phosphate solution to 4.7 with 0.2 M citric acid. Atropine standard solution was made by dissolving 1 mg of pure atropine in 10 mL of distilled water. The extract was dissolved in 2 N HCl, filtered, and washed with CHCl_3_ (three times) and neutralized with 0.1 N NaOH. 5 mL of BCG and 5 mL of phosphate buffer were added and completely extracted and diluted with chloroform. Accurately measured aliquots of atropine standard (0.4, 0.6, 0.8, 1, and 1.2 mL) were mixed with BCG and phosphate buffer, extracted with chloroform, and diluted with chloroform. The complex absorbance in chloroform was measured at 470 nm using a UV-Spectrophotometer. The blank was prepared similarly but without Atropine.

### 2.7 Molecular Study

#### 2.7.1 DNA Extraction

The NucleoSpin Plant II, Macherey-Nagel kit instructions were followed to extract DNA from the plant root sample (13817). The gel electrophoresis (1% w/v) method was used to ensure the purity of the extracted DNA and the same was documented using the Bio-Rad.

#### 2.7.2 Amplification

Master Mix Phire Plant (Thermo Scientific) and Applied Biosystems 2720 thermal cycler was used for the amplification of the primers selected (Table 1). PCR mix was prepared for the DNA sample along with a negative PCR control and positive control (certified reference material).

**Table 1:** Primers Used for PCR and Sequencing

Sr no	region of interest	name	seq (5′–3′)	bases	amplicon size (bp)
1	rbcLa	rbcLa-F	ATGATAACTCGACGGATCGC	20 bases	∼599
2		rbcLa-R	CTTGGATGTGGTAGCCGTTT	20 bases	
3	ITS	ITS1	TCCGTAGGTGAACCTGCGG	19 bases	∼400–600
4		ITS4	TCCTCCGCTTATTGATATGC	20 bases	
5	matK	matK413F1	TAATTTACAATCAATTCATTCAATATTTCC	30 bases	∼844
6		matK1257R1	GAAGAYCCACTATAATAATGAGAAAGATTT	30 bases	

ExoSAP-IT PCR Product Cleanup Reagent (Thermo Fisher) was used to clean the PCR product. Prior to sequencing, 2% agarose gel electrophoresis was used to validate the PCR product’s purity. As a molecular standard, a 100 bp DNA ladder (ExcelBand, SMOBIO) was employed. Using the BIO-RAD GelDoc-XR gel documentation system, gel images were captured.

#### 2.7.3 Sequencing

The DNA sequencing was performed with the PCR products purified with the ExoSAP. ABI BigDye Terminator v3.1 Cycle Sequencing reaction kit was used.

#### 2.7.4 DNA Sequence Analysis

The 3130 Genetic Analyzer Automated DNA Sequencing Machine were used to generate DNA sequences in.ab1 and FASTA formats, and Sequencing Analysis 5.1 software was used to do further analysis. A contig of the truncated sequence was created using forward and reverse strand sequences. Consequently, a single FASTA sequence was produced and further investigated. Using the nucleotide BLAST analysis tool, the sequencing similarity of the generated samples was examined with the sequences in the GenBank Database. The Clustal W alignment was utilized for multiple sequence alignment and comparing different sequences, and the degree of similarity between them was determined. For all three of the sample sequences, a phylogenetic tree was constructed using the MEGA 6 software utilizing the closest-matching source sequence data from the database (NCBI GenBank nucleotide sequence).

## 3 Results

### 3.1 Macroscopic Examination of the Root of *E. tithymaloides* (L.) Poit

**Figure 1:**
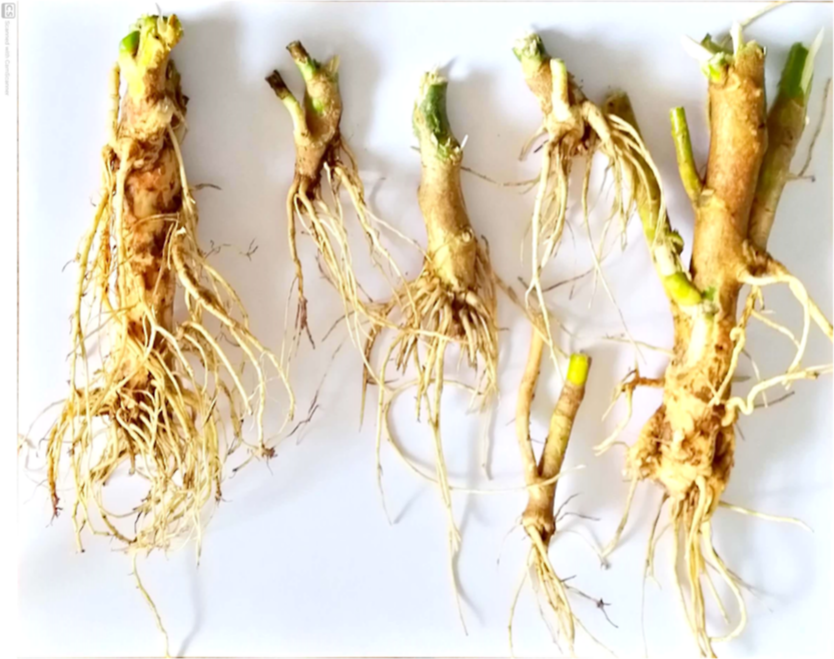
Macroscopic image of root of *Euphorbia tithymaloides* (L.) Poit.

The macroscopic characteristics of the roots as shown in Figure 1 were observed, including organoleptic characters, macro-morphological characteristics, and quantitative macroscopic measures (Table 2).

**Table 2:** Macroscopic Examination of the Root of *Euphorbia tithymaloides* (L.) Poit

Sr. no.	macroscopic characteristics	description
Organoleptic Properties		
1	color	
	upper/outer	light brown
	lower/inner	buff
2	texture	fibrous
3	odor	dusty
4	taste	slightly bitter
Macro-morphological Features		
5	type	tap
6	shape	cylindrical
7	length	7–10 cm
8	width	2–4 cm
9	surface	rough
10	fracture	fibrous

### 3.2 Powder Microscopy

**Figure 2:**
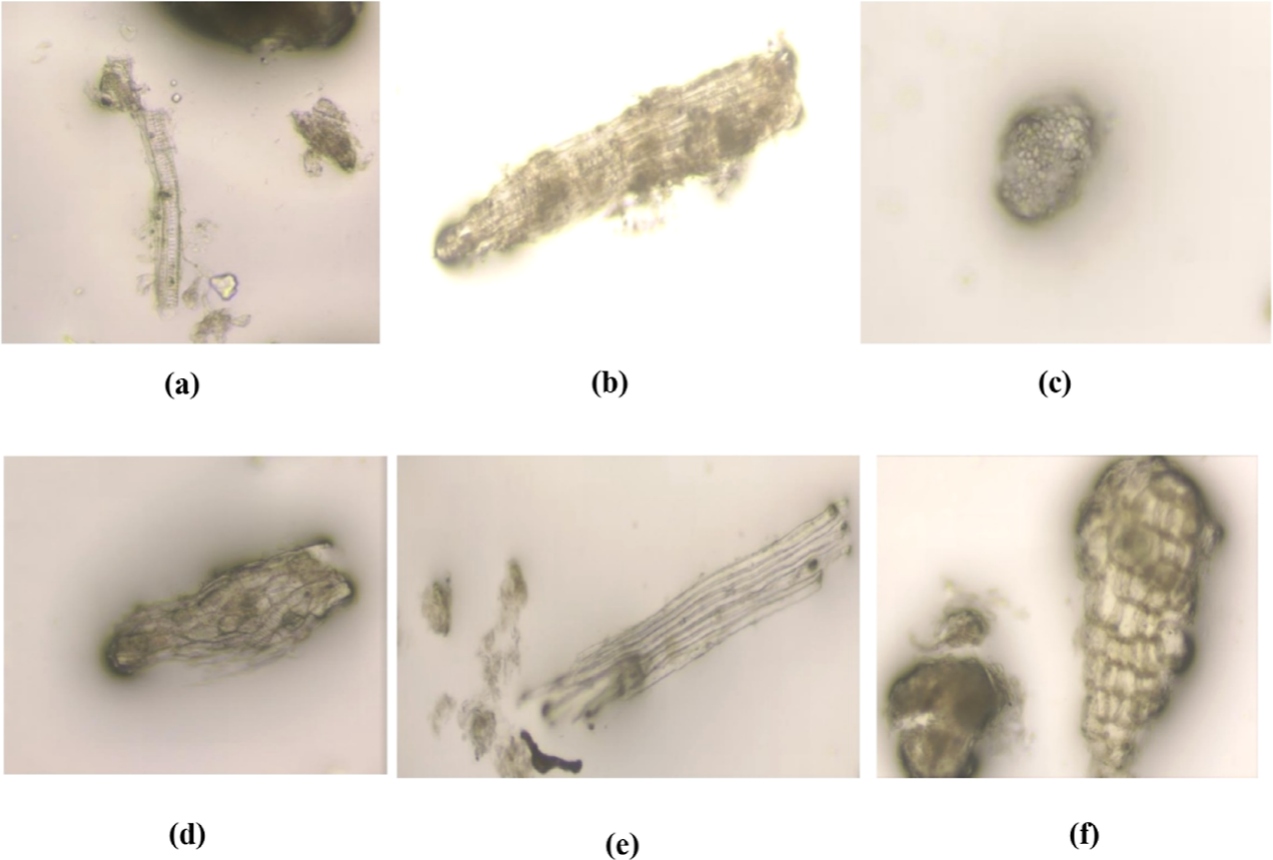
Microscopic images of (a) scalariform vessel; (b) medullary ray; (c) parenchymal cells containing starch; (d) sclereids; (e) fibre; and (f) cork.

Figure 2 depicts the numerous microscopic features of the root of *E. tithymaloides* (L.) Poit that are diagnostically important.

### 3.3 Analytical Standards of the Root of *E. tithymaloides* (L.) Poit

Total ash, water content, alcohol soluble extractive, water-soluble extractive value, acid insoluble ash value, soluble ash, and moisture content of the root of *E. tithymaloides* were determined and are reported in Table 3.

**Table 3:** Analytical Standards of the Root of *Euphorbia tithymaloides* (L.) Poit

Sr no	physiochemical parameters	% composition
1	total ash	7.5%
2	water soluble ash	2%
3	acid insoluble ash	3.5%
4	hexane soluble extractive value	2.60%
5	alcohol soluble extractive value	1.30%
6	hydro-alcoholic soluble extractive value	2.90%
7	moisture content	3.33%
8	foaming index	<100

### 3.4 Qualitative Phytochemical Analysis of the Crude Extract

The extracts were studied further to discover which phytochemical compounds were present. Flavonoid, alkaloid, terpenoids, steroid, tannin, and saponins are examples of common phytochemistry components found in the root extract (Table 4).

**Table 4:** Phytochemical Analysis of *Euphorbia tithymaloides* (L.) Poit Extracts^a^

phytochemicals	hexane extract	ethyl acetate extract	ethanol extract	ethanol/water extract
alkaloides	+	+	++	++
flavonoid	++	++	+	+
saponin	—	+	+	++
steroid and terpenoids	++	++	+	+
tannins	+	++	+	+

a — absent, + trace, ++ present, +++ concentrated.

### 3.5 Quantitative Phytochemical Analysis of the Crude Extract

#### 3.5.1 Total Flavonoid Content

**Figure 3:**
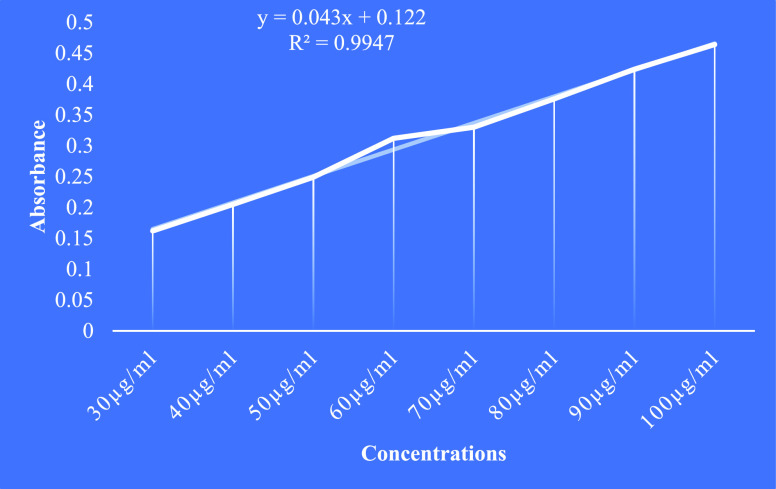
Calibration curve of quercetin.

The total flavonoid content was measured with all the extracts using quercetin as the standard. The calibration curve for quercetin was plotted as in Figure 3. The equation of the calibration curve of the quercetin standard was *y* = 0.043*x* + 0.122, *R*^2^ = 0.9947. Among the four crude extracts, ethyl acetate contained the highest amount of total flavonoid content compounds followed by hexane, ethanol, and then hydro alcoholic (Table 5).

**Table 5:** Quantitative Phytochemical Analysis of the Crude Extract

extract	flavonoid content (mg At E/g)	phenolic content (mg At E/g)	alkaloidal content (mg At E/g)
hexane	35.42667 ± 6.07	10.26667 ± 1.77	24.9945 ± 6.80
ethyl acetate	41.458 ± 1.33	7.115933 ± 0.36	37.93 ± 0.77
ethanol	21.69333 ± 1.48	6.3 ± 1.75	63.04 ± 3.08
ethanol/water	12.4333 ± 3.65	3.188167 ± 0.67	38.05 ± 0.93

#### 3.5.2 Total Phenolic Content

**Figure 4:**
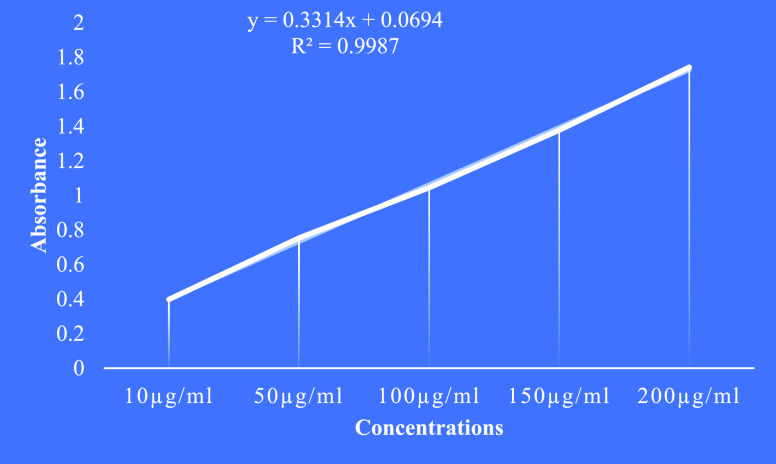
Calibration curve of gallic acid.

The total phenol concentration of four crude extracts evaluated using the FolinCiocalteu technique was reported as GAE. The gallic acid calibration curve revealed maximal absorbance at 765 nm (*y* = 0.3314*x* + 0.0694 *R*^2^ = 0.9987) (Figure 4). Among the four crude extracts, the hexane extract contained the highest total phenolic content compounds, followed by the ethyl acetate, ethanol, and hydro alcoholic extract content minimal (Table 5).

#### 3.5.3 Total Alkaloidal Content

**Figure 5:**
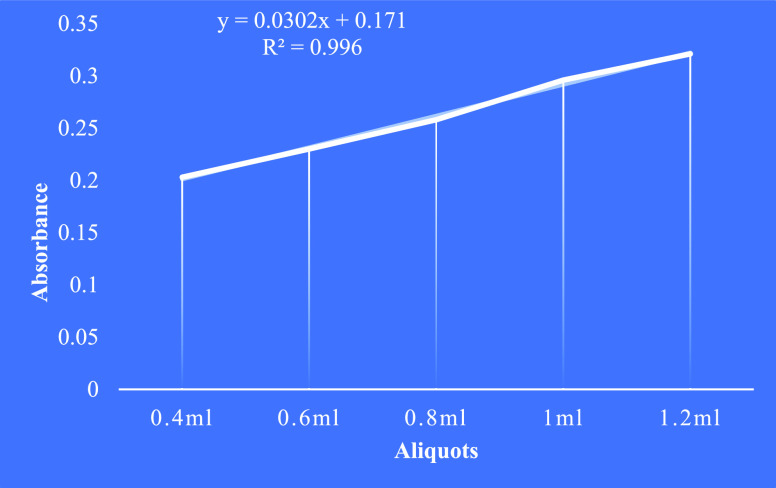
Calibration curve of atropine.

The total alkaloid content was determined using atropine as the reference standard. The calibration curve for the atropine was plotted with a maximum absorbance of 470 nm (*y* = 0.0302*x* + 0.171 R^2^ = 0.996) (Figure 5). Of all the root extracts, the ethanol extract contained a high amount of alkaloidal content followed by hydro alcohol, ethyl acetate, and hexane extracts (Table 5).

### 3.6 Molecular Study

**Figure 6:**
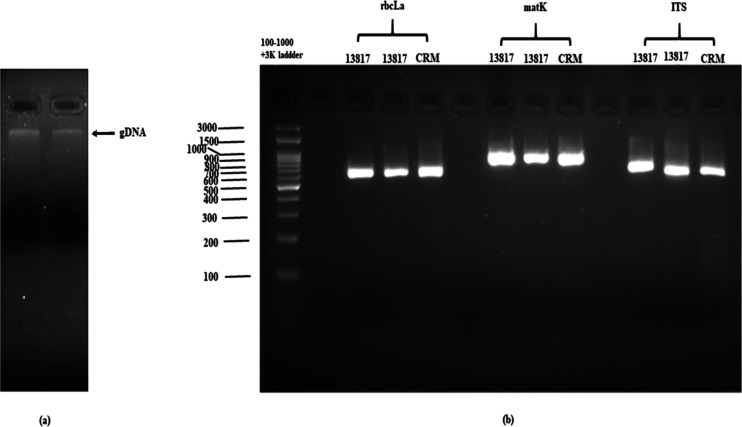
Gel electrophoresis: (a) quality of the extracted DNA: lane 1 and 2—extracted DNA of sample ID 13817 (1% w/v); (b) 2% agarose gel of control, PCR product of samples and marker: lane 1: 100–1000 + 3k bp DNA marker; lanes 2, 6, 10: NTC (negative test control), lanes 3–4: 13817 sample PCR product (rbcL). Lanes 7–8: 13817 sample PCR product (matK). Lanes 11–12: 13817 sample PCR Product (ITS), lanes 5,9,13- certified reference material (CRM) or standard positive control (rbcLa, matK, and ITS) respectively.

The DNA from the root was extracted, and its purity was ensured by gel electrophoresis (Figure 6). To determine the quality and quantity of the extracted DNA, the samples were spectrophotometrically observed at 260 and 280 nm along with their ratios. The absorbance at 260 nm was found to be 4.037 and its concentration was determined to be 201.84 ng/μL. Likewise, the absorbance at 280 nm was found to be 2.145, whereas the 260/280 ratio was 1.88, which lies close to the ideal value 1.8. The 260/230 ratio of 1.89 suggests that there may be some contamination in the DNA sample. A ratio below the ideal value of 2.0 indicates the presence of few contaminants that may interfere with downstream applications. The rbcL loci primers selected were rbcLa-F and rbcLa-R, and the amplicon size was ∼599 bp. Similarly, the MatK primers MatK413F1 and MatK1257R1 weighed ∼844 bp and the ITS primers ITS1 and ITS4 weighed ∼400–600 bp. The forward and reverse sequences were generated, and a contig of the trimmed sequence was generated. Using BLAST, the generated sequences were compared to the GenBank database. The sequence alignment was performed using the Clustal W algorithm, and the analysis revealed the maximum number of matches with a high percentage. The *Pedilanthus tithymaloides* chloroplast rbcL gene (AB267959.1) showed a maximum similarity of 99.83 (Table 6).

**Table 6:** BLAST Analysis of rbcLa

description	max score	total score	query cover (%)	*E* value	per. ident	per. ident
P. tithymaloides chloroplast rbcL gene for ribulose-1,5-bisphosphate carboxylase/oxygenase large subunit, partial cds	1051	1051	94	0	99.83	AB267959.1
Euphorbia tirucalli voucher N.Wei 1064 (HIB) chloroplast, complete genome	1044	1044	96	0	98.81	MT395048.1
Euphorbia enterophora voucher N.Wei 1044 (HIB) chloroplast, complete genome	1044	1044	96	0	98.81	MT395033.1
Euphorbia milii chloroplast, complete genome	1038	1038	96	0	98.64	MN713924.1
Euphorbia larica chloroplast, complete genome	1038	1038	96	0	98.64	MN646683.1

**Figure 7:**
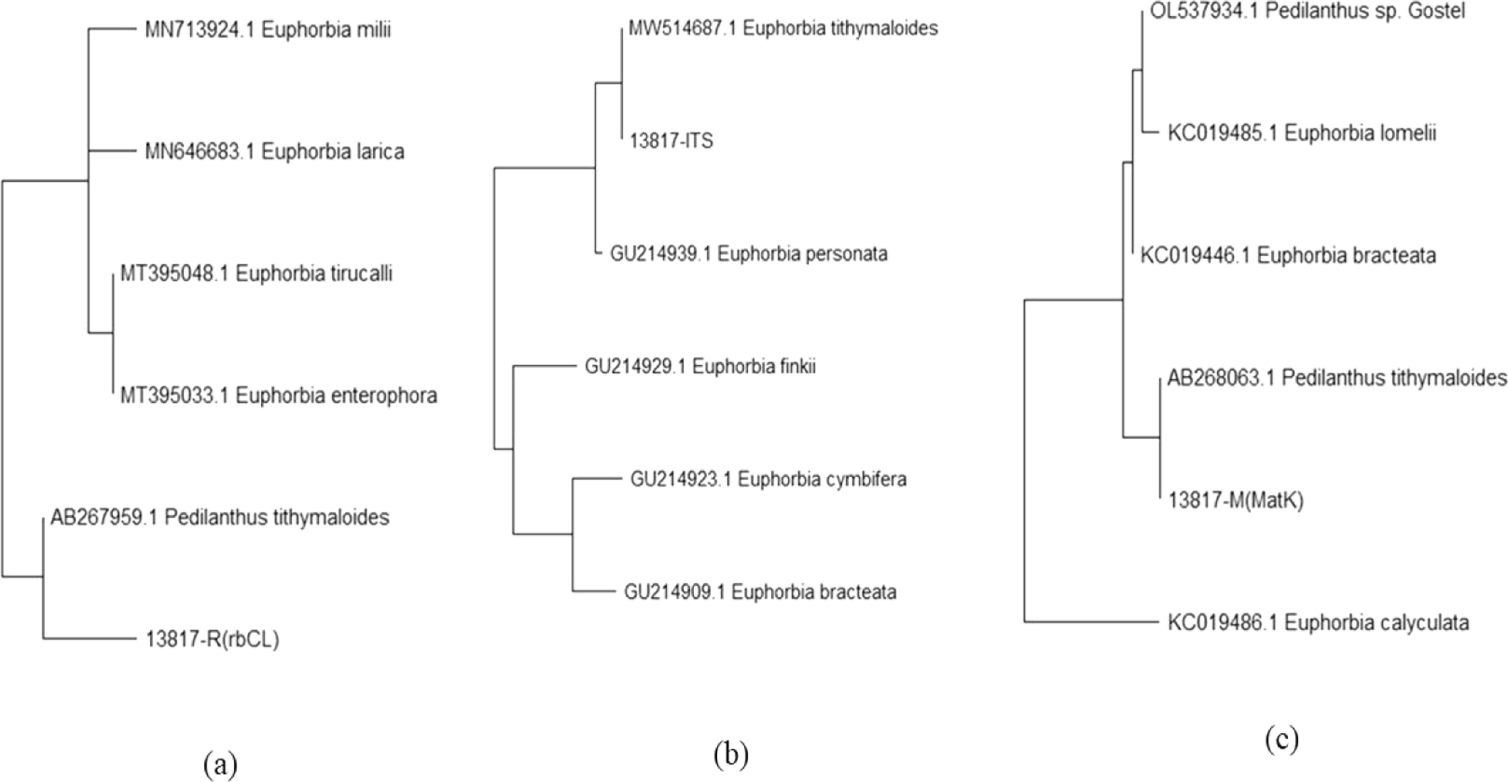
Phylogenetic tree drawn with first five hits in BLAST analysis: (a) rbcL; (b) ITS; and (c) matK.

The neighbor-joining approach was used to infer the evolutionary history.^27^ The ideal tree is displayed, with a branch length total of 0.04366671. In the bootstrap test (500 repetitions), the proportion of duplicate trees in which the linked taxa grouped together is displayed next to the branches.^28^ The tree is rendered to scale with branch lengths in the same units as the evolutionary distances used to estimate the phylogenetic tree. The evolutionary distances, which are measured in terms of the number of base substitutions per site, were calculated using the maximum composite likelihood technique.^29^ Six nucleotide sequences were subject to this investigation. Codon positions 1st + 2nd + 3rd + noncoding were included. For each sequence pair, all uncertain positions were eliminated (pairwise deletion option). The final dataset had 671 positions altogether. In MEGA X, evolutionary studies were carried out.^30^ The closest plant species was used to draw the phylogenetic tree for the matches in the BLAST data (Figure 7). It displays the amount of base substitutions made at each place between sequences. The Maximum Composite Likelihood model was used for the analyses.^29^ Six nucleotide sequences were subject to this investigation. Codon positions 1st + 2nd + 3rd + Noncoding were included. For each sequence pair, all uncertain positions were eliminated (pairwise deletion option). The final dataset had 671 locations altogether. In MEGA X, evolutionary studies were performed.^30^ From the BLAST hits, the distance matrix showed the closest distance between the nearby species (Table 7).

**Table 7:** Estimates of Evolutionary Divergence between Sequences (rbcl)

MN713924.1_Euphorbia_milii					
MN646683.1_Euphorbia_larica	0.00686				
MT395048.1_Euphorbia_tirucalli	0.00514	0.00513			
MT395033.1_Euphorbia_enterophora	0.00514	0.00513	0.00000		
AB267959.1_Pedilanthus_ tithymaloides	0.01055	0.01054	0.00878	0.00878	
13817_R_rbCL	0.01904	0.01902	0.01728	0.01728	0.00526

Likewise, the blast analysis of the ITS region revealed a 99.84% match with *E. tithymaloides* voucher BGK1984-2163 (Kew) (Table 8). The phylogenetic tree was plotted with the closest species of *E. tithymaloides*, and the distance matrix was determined (Table 9). The finally generated sequence with MatK was submitted to BLAST analysis, which showed a high percentage of 100% with the *P. tithymaloides* matK loci. The matK loci had the closest and highest similarity of 100% (Table 10), followed by other *Euphorbia* species with the least, at 95.11%. Likewise, the phylogenetic tree and distance matrix of the species closest to *E. tithymaloides* were plotted (Table 11).

**Table 8:** BLAST Analysis of ITS

description	max score	total score	query cover (%)	*E* value	per. ident	description
Euphorbia tithymaloides voucher BGK1984-2163 (Kew)	1123	1123	100	0	99.84	MW514687.1
Euphorbia personata voucher MEXU/MEO & NIC 955	1096	1096	100	0	99.02	GU214939.1
Euphorbia finkii voucher MEXU/MEO & NIC 917	985	985	99	0	95.89	GU214929.1
Euphorbia cymbifera voucher MEXU/MEO & NIC 979	974	974	100	0	95.43	GU214923.1
Euphorbia bracteata voucher MEXU/MEO & NIC 845	965	965	100	0	95.11	GU214909.1

**Table 9:** Estimates of Evolutionary Divergence between Sequences (ITS)

MW514687.1_Euphorbia_tithymaloides 13817_ITS	0.00000				
GU214939.1_Euphorbia_personata	0.00533	0.00659			
GU214923.1_Euphorbia_cymbifera	0.03825	0.04418	0.04063		
GU214909.1_Euphorbia_bracteata	0.03836	0.04776	0.04074	0.01820	
GU214929.1_Euphorbia_finkii	0.03270	0.03747	0.03565	0.03227	0.02876

**Table 10:** BLAST Analysis of MatK

description	max score	total score	query cover (%)	*E*-value	per. ident	accession
P. tithymaloides chloroplast matK gene for maturaseK, partial cds	1445	1445	100	0	100	AB268063.1
Euphorbia bracteata voucher Berry, P.E. 7839 (MICH) maturaseK (trnK) gene, complete cds; chloroplast	1417	1417	100	0	99.36	KC019446.1
Pedilanthus sp. Gostel 565 voucher BRIT/Gostel 565 maturaseK (matK) gene, partial cds; chloroplast	1411	1411	100	0	99.23	OL537934.1
Euphorbia lomelii voucher Van Devender, T.R. 2007–1105 (ASDM) maturaseK (trnK) gene, complete cds; chloroplast	1400	1400	100	0	98.98	KC019485.1
Euphorbia calyculata voucher Steinmann, V.W. 3472 (IEB) maturaseK (trnK) gene, complete cds; chloroplast	1284	1284	100	0	96.29	KC019486.1

**Table 11:** Estimates of Evolutionary Divergence between Sequences (matK)

AB268063.1_Pedilanthus_tithymaloides					
13817-M_MatK	0.00000				
OL537934.1_Pedilanthus_sp._Gostel	0.00778	0.00778			
KC019485.1_Euphorbia_lomelii	0.01039	0.01039	0.00256		
KC019446.1_Euphorbia_bracteata	0.00648	0.00648	0.00128	0.00385	
KC019486.1_Euphorbia_calyculata	0.04045	0.04045	0.03724	0.04007	0.03583

#### 3.6.1 rbcLa Analysis

a DNA sequencing

The following sequences were generated for the sample (13817-R(rbcl)):

**>13817-R(rbCL-F)** ATATTGGATCAAGCTGGTGTTAAGATTATAAATTGACTTATTATACTCCTGAATATGAAACCAAAGATACTGATATCTTGGCAGCATTCCGAGTAACTCCTCAACCTGGAGTTCCACCTGAGGAAGCAGGAGCTGCCGTAGCTGCTGAATCTTCTACTGGTACATGGACACTGTGTGGACCGATGGGCTTACCAGTCTTGATCGTTATAAAGGACGATGCTACCACATCGAGCCCGTTGCTGGAGAAGAAAATCAATATATTGCTTATGTAGCTTACCCCTTAGACCTTTTTGAAGAAGGTTCTGTTACTAACATGTTTACCTCCATTGTGGGTAATGTATTTGGGTTCAAAGCCCTACGCGCTCTACGTCTGGAGGATTTACGAATCCCTACTTCTTATACTAAAACTTTCCAAGGGCCACCTCATGGCATCCAAGTTGAGAGAGATAAATTGACAAATATGGTCGCCCTCTATTGGGTTGTACTATTAAACCAAAATTGGGGCTATCCGCTAGAATTACGGTAGAGCGGTTTATGAATGTCTTCGCGGTGGATTGAATTATTTCCAGA.

**>13817-R(rbCL-R)** TAAGGCAACCCCAAAACAGAGACTAAAGCAAGTGTTGGATTCAAGGCTGGTGTTAAAGATTATAAATTGACTTATTATACTCCTGAATATGAAACCAAAGATACTGATATCTTGGCAGCATTCCGAGTAACTCCTCAACCTGGAGTTCCACCTGAGGAAGCAGGAGCTGCCGTAGCTGCTGAATCTTCTACTGGTACATGGACAACTGTGTGGACCGATGGGCTTACCAGTCTTGATCGTATAAAGGACGATGCTACCACATCGAGCCCGTTGCTGGAGAAGAAAATCAATATATTGCTTATGTAGCTTACCCCTTAGACCTTTTTGAAGAAGGTTCTGTTACTAACATGTTTACCTCCATTGTGGGTAATGTATTTGGGTTCAAAGCCCTACGCGCTCTACGTCTGGAGGATTTACGAATCCCTACTTCTTATACTAAAACTTTCCAAGGGCCACCTCATGGCATCCAAGTTGAGAGAGATAAATTGAACAAATATGGTCGCCCTCTATTGGGTTGTACTATTAAACCAAAATTGGGGCTATCCGCTAAGAATACGTA GAGCGTA.

**>13817-R(rbCL) (assembled contig)** TAAGGCAACCCCAAAACAGAGACTAAAGCAAGTGTTGGATTCAAGGCTGGTGTTAAAGATTATAAATTGACTTATTATACTCCTGAATATGAAACCAAAGATACTGATATCTTGGCAGCATTCCGAGTAACTCCTCAACCTGGAGTTCCACCTGAGGAAGCAGGAGCTGCCGTAGCTGCTGAATCTTCTACTGGTACATGGACAACTGTGTGGACCGATGGGCTTACCAGTCTTGATCGTTATAAAGGACGATGCTACCACATCGAGCCCGTTGCTGGAGAAGAAAATCAATATATTGCTTATGTAGCTTACCCCTTAGACCTTTTTGAAGAAGGTTCTGTTACTAACATGTTTACCTCCATTGTGGGTAATGTATTTGGGTTCAAAGCCCTACGCGCTCTACGTCTGGAGGATTTACGAATCCCTACTTCTTATACTAAAACTTTCCAAGGGCCACCTCATGGCATCCAAGTTGAGAGAGATAAATTGAACAAATATGGTCGCCCTCTATTGGGTTGTACTATTAAACCAAAATTGGGGCTATCCGCTAAGAATTACGGTAGAGCGGTTTATGAATGTCTTCGCGGTGGATTGAATTATTTCCAGA.

b BLAST analysis

Library details: molecule type: DNA, query length: 607 bases database name: nr.

Description: nucleotide collection (nt) *program: BLASTN 2.2.28+.

c Distance matrix

#### 3.6.2 ITS Analysis

a DNA sequencing.

The following sequences were generated for the sample (13817—ITS).

**>13817(ITS1)** GGGCCGTAGCTACTGCAACGACCCGTGACATGTTCATAAACATGGGTGCCGGTGCGGGATTCGTCCGGCAACGGCATCCCACATGGGCCGGGCAGCGGGGACGCGGGTGGTGCACCCCGTGTTCTCCTTGTCCGGTTCCTTCTAACCAAACACCGACGCCAAACGCGTCAAGGAACTGCGAAAAAAAGGCAGCTTAGGCCCCGGAAACGGCGGTAACCAATGCTGTTTTGGAATAAAAACGACTCTCGGCAACGGATATCTCGGCTCTCGCATCGATGAAGAACGCAGCGAAATGCGATACTTGGTGTGAATTGCAGGATCCCGCGAACCATCGAGTCTTTGAACGCAAGTTGCGCCCGAAGCCTTTCGGCCGAGGGCACGCCTGCCCTGGGTGTCACTCAACTGTCGCCCCGACCCCCTCCTGAAAGGAGGGACGTGAGGGGCGGATGATGGCTTCCCGTGAGCTTTGCAGCCCGCGGTTGGCCCAAAATTCTGGTCCCTGGAACAGAATCCACGGCAATCGGTGGGTTGAAAGACCCTCGTAAAATGTCGTGGTTTCACGGAAGCCAGAGGCGGCAATTAGACCCCAGAGCGCATCCCAAAGCAGCGCTCGAACGGCGACCCCAGGTCAGGCGGGATTACCCGCTGAGTTTAAGCTTCGAGGGGGGGGGGGAGAGAAAAAA.

**>13817(ITS4)** CTTTTTTTCGGCAGTTCCCGACGCCTTGGGTCGGGTTGGTAGAGACCGACAAGAGGAACACGGTGCACACCGGTCCCGCGCCCGCCCTTGGAGCCGTGCCGGACGATCCCGACCGGAAGCCAGTTTATGAAATGTCCCGGGTCGTTTGCGGGCGGGCCTGACAATGATCCTTTCGTAAGGGGGGGGCTGCGAAGGATCATGTCGAGGCCTGCCTAGCAAAACGACCCGTGAACATGTTCATAAACATGGCTGCCGGTGCGGGATTCGTCCGGCAACGGCATCCCCACATTGGCCGGGCAGCGGGGACGCGGGTGGTGCACCCCCGTGTTCTCCTTGTCCGGTTCCTTCTAACCCAAACACCGACGCCAAACGCGTCAAGGAACTGCGAAAAAAAGGCAGCTTAGGCCCCGGAAACGGCGGTAACCAATGCTGTTTTGGAATAAAAACGACTCTCGGCAACGGATATCTCGGCTCTCGCATGATGAAGAACGCAGCGAAATGCGATACTTGGTGTGAATTGCAGGATCCCGCGAACCATCGAGTCTTTGAACGCAAGTTGCGCCCGAAGCCTTTCGGCCGAGGGCACGCCTGCCTGGGTGTCACTCAACTGTCGCCCCGACCCCCTCCTGAAAGGAGGGACGTGAGGGGCGGATGATGGCTTCCCGTGAGCTTTGCAGCCCGCGGTTGGCCCAAAATTCTGGTCCCTGGAACAGAATCCACGGCAATCGGTGGTTGAAAGACCCTCGTAAAATGTCGTGGTTTCACGGAAGCCAGAGGCGGCAATTAGACCCCAGAGCGCATCCAAAGCAGCGCTCGAACGGCGACCCCAGTCAGGCGGATAGACCA.

**>13817-ITS(assembled contig)** GGGTGCCGGTGCGGGATTCGTCCGGCAACGGCATCCCACATGGGCCGGGCAGCGGGGACGCGGGTGGTGCACCCCGTGTTCTCCTTGTCCGGTTCCTTCTAACCAAACACCGACGCCAAACGCGTCAAGGAACTGCGAAAAAAAGGCAGCTTAGGCCCCGGAAACGGCGGTAACCAATGCTGTTTTGGAATAAAAACGACTCTCGGCAACGGATATCTCGGCTCTCGCATCGATGAAGAACGCAGCGAAATGCGATACTTGGTGTGAATTGCAGGATCCCGCGAACCATCGAGTCTTTGAACGCAAGTTGCGCCCGAAGCCTTTCGGCCGAGGGCACGCCTGCCCTGGGTGTCACTCAACTGTCGCCCCGACCCCCTCCTGAAAGGAGGGACGTGAGGGGCGGATGATGGCTTCCCGTGCGCTTTGCAGCCCGCGGTTGGCCCAAAATTCTGGTCCCTGGAACAGAATCCACGGCAATCGGTGGTTGAAAGACCCTCGTAAAATGTCGTGGTTTCACGGAAGCCAGAGGCGGCAATTAGACCCCAGAGCGCATCCAAAGCAGCGCTCGAACGGCGACCCCAGGTCAGGCGGGATTACCCGCTGAGTTTAAGC.

b BLAST analysis.

Library details: molecule type: DNA, query length: 612 bases database name: nr.

Description: nucleotide collection (nt) *program: BLASTN 2.2.28+.

c Distance matrix.

#### 3.6.3 matK Analysis

a DNA sequencing.

The following sequences were generated for the sample (13817M(matK)):

**>13817-M(MatK-F)** ATAAGAGAAATTTCCGCATTTAATTATGTATCAGATGTATTAATACCTTATCCCATCCATCTTGAAAAATTGGTCCAAACCCTTCGTTTTTGGGTGACAGACCCTTCTTCTTTGCATTTTTTACGATTCTTTCTTCATCAGTATTGGAATTGGAACAGTCTTATTATTCCAAAGAAATCAATTTCGATTTTTCGAAAAAATAATCCACGATTTTTCTTGTTCATATATAATATTCATATATATCAATATGAATCCATCTTCTTTTTTCTTCGTAATCAGTCCTTTCATTTACGATCAACATTTTCTCGAGTCTTTCTTGAACGAATTTTTTTCTATGGAAAACTAGAACATTTTGCAGAAGTTTTTGCTAATGATTTTCAGACCATCCTAGGGTTGTTCAAGGAGCCTTTCATGCATTATGTTAGATATCAAGGAAAATCAATTCTGGCTTTAAAAGATAAGCCCTTTCTGATGAAAAAATGGAAATATTACCTTGTCAATTTATGTCAATGTCATTTTTATGTGTGGTTTCAACCAGAAAAGATCTATATCAATTCATTATCCAAAAATTCTCTCTATTTTGGGGGATATCTTTCAAGTGTACAAATCAATCCTTTGGTAGTACGGAGTCAAATGCTAGAAAATTCATATCTATAGCTAACGATGATACTATGAAGAAACTCGATACAATAGTTCCAATTACTCCTTTAATTAGATTATTGGCAAAATGCAATTTTGTAATGCAGTAGACATCCTATTAGTAAACCGATCCGGGCTCATTCATCCGATTCAGATATTATCGAACAATTTTGTGCGTATATGCAGAAATCTTTCTCATTATCTCGGGGGGGGGACTCACCAAAAA.

**>13817-M(MatK-R)** CATCATCAAATATTTCCTTTTTAGAGGACAAATTTCCGCATTAATTATGTATCAGATGTACTAATACTATCCCATCCATCTTGAAAAATGGTCCAAACCCTTCGTTTTTGGGTGACAGACCATCTTCTTGCATTTTTTACGATTCTTCTTCATCAGTATTGGAATTGGAACAGTCTTATTATTCCAAAGAAATCAAATTTCGATTTTTCGAAAAAATAATCCACGATTTTTCTTGTTCATATATAATATTCATATATATCAATATGAATCCATCTTCTTTTTCTTCGTAATCAGTCCTTTCATTTACGATCAACATTTTCTCGAGTCTTTCTTGAACGAATTTTTTTCTATGGAAAACTAGAACATTTTGCAGAAGTTTTTGCTAATGATTTTCAGACCATCCTAGGGTTGTTCAAGGAGCCTTTCATGCATTATGTTAGATATCAAGGAAAATCAATTCTGGCTTTAAAAGATAAGCCCTTTCTGATGAAAAAATGGAAATATTACCTTGTCAATTTATGTCAATGTCATTTTTATGTGTGGTTTCAACCAGAAAAGATCTATATCAATTCATTATCCAAAAATTCTCTCTATTTTGGGGGATATCTTTCAAGTGTACAAATCAATCCTTTGGTAGTACGGAGTCAAATGCTAGAAAATTCATATCTAATAGCTAACGATAATACTATGAAGAAACTCGATACAATAGTTCCAATTACTCCTTTAATTAGATTATTGGCAAAAATGCAATTTTGTAATGCAGTAGGACATCCTATTAGTAAACCGATCCGGGCTCATTCATCCGATCAGATATATCGACAAATTTTGCCTATAATTCC.

**>13817-M(MatK)(assembled contig)** TTATGTATCAGATGTATTAATACCTTATCCCATCCATCTTGAAAAATTGGTCCAAACCCTTCGTTTTTGGGTGACAGACCCTTCTTCTTTGCATTTTTTACGATTCTTTCTTCATCAGTATTGGAATTGGAACAGTCTTATTATTCCAAAGAAATCAATTTCGATTTTTCGAAAAAATAATCCACGATTTTTCTTGTTCATATATAATATTCATATATATCAATATGAATCCATCTTCTTTTTTCTTCGTAATCAGTCCTTTCATTTACGATCAACATTTTCTCGAGTCTTTCTTGAACGAATTTTTTTCTATGGAAAACTAGAACATTTTGCAGAAGTTTTTGCTAATGATTTTCAGACCATCCTAGGGTTGTTCAAGGAGCCTTTCATGCATTATGTTAGATATCAAGGAAAATCAATTCTGGCTTTAAAAGATAAGCCCTTTCTGATGAAAAAATGGAAATATTACCTTGTCAATTTATGTCAATGTCATTTTTATGTGTGGTTTCAACCAGAAAAGATCTATATCAATTCATTATCCAAAAATTCTCTCTATTTTGGGGGATATCTTTCAAGTGTACAAATCAATCCTTTGGTAGTACGGAGTCAAATGCTAGAAAATTCATATCTAATAGCTAACGATAATACTATGAAGAAACTCGATACAATAGTTCCAATTACTCCTTTAATTAGATTATTGGCAAAAATGCAATTTTGTAATGCAGTAGGACATCCTATTAGTAAACCGATCCGGGCTCATTCATCCGATTCAGATATTATCG.

b BLAST analysis.

Library details: molecule type: DNA, query length: 728 bases database name: nr description: nucleotide collection (nt) *program: BLASTN 2.2.28+.

c Distance matrix.

## 4 Discussion

A pharmacognostic study of a plant involves the scientific examination of the plant’s physical and chemical characteristics, as well as its traditional uses. The goal of such a study is to establish a set of quality standards for the plant material that can be used to ensure its authenticity and purity. One such plant, *E. tithymaloides*, was selected for the pharmacognostic study. Primarily, the macroscopic analysis of plants aids in identifying authentic materials. The *E. tithymaloides* root is macroscopically seen to be light brown on the outside and buff color on the inside, with a fibrous texture and dusty and mildly bitter flavors. Similar to this, one of the essential factors in pharmacopeia is the study of powder microscopy. The powdered roots of *E. tithymaloides* revealed the presence of scalariform vessels with a pitted bordered wall, radially cut medullary rays, a group of fragmented oval-shaped parenchyma cells with a thin wall containing starch, a thick wall with an oval to rectangular-shaped cork, group of sclereids with polygonal wall, elongated, blunt end, and thin wall fibers.

Preliminary phytochemical investigation of medicinal plants is the initial step in the process of identifying and characterizing the phytoconstituents present in a plant. The root powder was subjected to extraction with solvents such as ethanol, ethanol: water, hexane, and ethyl acetate. These extracts were then used for the physiochemical and phytochemical analysis, which indicated the presence of alkaloids, tannins, flavonoids, steroids, terpenoids, and traces of saponins. A similar study was conducted by Matisui et al. in 2017, where the phytochemical analysis was performed with the leaves of *E. tithymaloides*. It was reported that the ethanol, ethyl acetate, and hexane extracts showed the presence of steroids, triterpenes, saponins, tannin, and coumarin. It was observed that the leaves lack alkaloids, which makes the root of *E. tithymaloides* more advantageous for therapeutic use than the leaves.^31^

Extractive values are a measure of the amount of certain active or inert ingredients present in a drug. They are used to determine the purity and potency of a drug and can help detect if a drug is exhausted or adulterated. The United States Pharmacopeia (USP) and the European Pharmacopeia (EP) provide guidelines for the acceptable range of extractive values for various drugs. Drugs that fall outside of these ranges may be considered exhausted or adulterated and should not be used. The total ash content of the root of *E. tithymaloides* was found to be 7.5%, indicating the total amount of inorganic matter present in the plant. The water-soluble ash value of 2% revealed the presence of water-soluble compounds such as inorganic compounds, acids, and sugars. The hexane, alcohol, and hydro-alcohol soluble extractive values show the presence of polar-soluble solvents such as tannins, flavonoids, and alkaloids. Similarly, the foaming index and moisture content were both found to be <100 and 3.33%, indicating that no foaming agents and less moisture were present in the root samples. The presence of moisture in the plant material can have a significant impact on the quality and stability of the phytoconstituents present in the material. Moisture serves as an ideal medium for the growth of bacteria and fungi, which can lead to the degradation of the plant material and the loss of its medicinal properties.^32,33^ Additionally, moisture can also cause the hydrolysis and oxidation of moisture-sensitive phytoconstituents, such as alkaloids, flavonoids, and terpenoids, which can result in a decrease in their concentration and effectiveness. These findings agree with the phytochemical analysis, which shows the presence of polar-soluble solvents and traces of saponins.

The primary goal of quantitative chemical analysis is to estimate the amounts of the plant’s major phytoconstituents classes. Flavonoids, phenols, and alkaloids are three important classes of phytoconstituents that are commonly found in medicinal plants. They are known for their medicinal properties and have been used in traditional medicine for centuries. Their presence in medicinal plants is often used as a measure of the plant’s quality and potency.^34^ This study examined the total amount of flavonoids, phenols, and alkaloids. The aluminum chloride colorimetric test was used to evaluate the sample’s total flavonoid content. The assay revealed that the ethyl acetate extract contains the highest amount of flavonoids, while the hydro-alcoholic extract showed the lowest amount. The ethyl acetate extract’s high concentration of flavonoids is comparable to the research conducted by Chávez et al. in 2022. It was reported that, when compared to hexane, water, and dichloromethane, the ethyl acetate extract of *E. tithymaloides* leaves contained a high amount of flavonoids.^35^ This indicates that *E. tithymaloides* in general, as a whole plant, possesses a good amount of flavonoids. Likewise, using gallic acid as a reference, the Folin–Ciocalteu method was used to quantify the total phenol concentration of the root extracts. It was clear from the assay that the hexane extracts had a higher phenolic content than other extracts. The total alkaloidal content assay showed that the ethanol extract had a high amount of alkaloids.

The nuclear ribosomal DNA ITS regions ITS1 and ITS2 and the chloroplast genes matK, rbcL, and trnH-psbA are the markers in plant barcoding that are most often investigated. These markers are highly informative for identifying and differentiating plant species. Recently, the use of DNA barcoding based on the markers rbcl, ITS, and MatK has gained significant traction in the field of plant species authentication.^36^ In this study, all three markers were used for the identification of *E. tithymaloides*. The sequence match with rbcl loci showed a match of 98.64 to 99.83% with the top five hits from the BLAST analysis. Similarly, the sequence match with the MatK loci was from 96.25 to 100%. These results are in contrast with the phylogenetic study conducted by El-Banhawy, 2020 in the genus Euphorbia.^37^ It was reported that the rbcl was the least successful and matK genes were not significantly different in identifying the Egyptian Euphorbia. However, the results of this study reveal a good identity score of 99.84 and 100 for *Euphorbia* plant grown in India. The difference in the identification of the genus using the rbcl and matk loci could be varied due to the geographical location and environment of the plant growth. These genetic and geography-based changes and their identification by rbcl and ITS were expressed by Shawkat and Ahmed, 2022 in a comparative study. It was reported that the rbcl and ITS2 were able to provide a good resolution among the *Euphorbia tirucalli*, *Euphorbia hirta*, and *Euphorbia peplus* but showed only a minor change in the evolution taxa and phylogenetic tree.^39^ These data are comparable to the results observed in this study as the phylogenetic tree and the distance matrix shows a minor change and were able to identify the closest species with a good matching percentage. The ITS is considered to be one of the most informative markers for species identification in plants, as it has a high degree of variation among different plant species. The ITS region is located between the 18S and 28S rRNA coding regions and is transcribed as part of the rRNA precursor molecule. The ITS region is highly variable, containing both coding and non-coding regions, which makes it a valuable marker for species identification. Additionally, the ITS region is present in multiple copies in the genome, which increases the chances of detecting variations among different species.^38^ Likewise, the ITS primers ITS1 and ITS4 in this study revealed a good match from 95.11 to 99.84% from the BLAST analysis. The ITS loci results are comparable with the study experimented on by Kim et al., in 2020 where DNA barcoding was performed for Korean Euphorbiaceae. It was reported that among the rbcl, ITS, and MatK loci, the ITS was the most beneficial and can be used to identify other Korean Euphorbiaceae plants using ITS as a single barcode.^40^ Likewise, the results from this study show that all the three loci were able to efficiently identify the *E. tithymaloides*. In addition, the 100% true match with the MatK loci shows that it can be used as a standalone to identify *E. tithymaloides* from the subspecies and other Euphorbiaceae plants as well.

One of the main reasons for the adulteration and substitution of *E. tithymaloides* is the high demand for the plant and its medicinal properties. This has led to the collection of the plant from wild populations, which can lead to over-harvesting and depletion of wild populations. Another reason is that *E. tithymaloides* is often confused with closely related species, and this can lead to misidentification and substitution. For example, *E. tithymaloides* is often confused with *Euphorbia lathyris*, which is not used for medicinal purposes and is considered toxic. Adulteration and substitution can have serious consequences for both the consumers and the environment. Consumers may be unknowingly consuming harmful substances or receiving ineffective treatment, while wild populations of medicinal plants may become endangered due to over-harvesting. To address these issues, it is important to establish and use DNA barcoding techniques for the identification and authentication of medicinal plants, including *E. tithymaloides*, to ensure that the plant material being sold is authentic and unadulterated. Additionally, conservation measures should be implemented to protect wild populations of medicinal plants from over-harvesting, and regulations should be put in place to control the trade of medicinal plants.

## 5 Conclusions

The conventional and molecular pharmacognostic study on the root of *E. tithymaloides* has been carried out in this study and reported for the first time. The results from the micro-and macroscopic studies, phytochemical analysis, and DNA barcoding had shown significant results in identifying the *E. tithymaloides*. It is important to keep in mind that DNA barcoding is one tool among many that can be used to identify and authenticate medicinal plants and it should be used in combination with other methods, such as morphological and chemical analysis, to ensure accurate identification. The results of this pharmacognostical study can be used to establish a set of quality standards for the *E. tithymaloides*, which can be used to ensure its authenticity and purity. This is important for ensuring the safety and efficacy of traditional medicine and medicinal products made from *E. tithymaloides*.
